# Complete genome sequence of the *Microbacterium foliorum* bacteriophage Garey24

**DOI:** 10.1128/mra.01215-23

**Published:** 2024-02-05

**Authors:** Matías R. Migueletti, Julieta García Rey, Josefina Micheloni, Camila Lomanto, Elisa Martelli, Gastón Sánchez, Julián M. Colombo, Luciano M. Vallecillo, Francisco Lamagni, Tomás Giusti, Fabrina Acosta, Franco Villagrán, Martín Gvozdenovich, Abril Pricco Frakich, Tulio Pianesi, Gonzalo Tulin, Florencia C. Mascali, Tomás D. Petitti, Mariano A. Torres Manno, Corina M. Fusari, Laura Buttigliero, María Florencia Giordana, Hugo Gramajo, Lautaro Diacovich, Martín Espariz, María Alejandra Mussi

**Affiliations:** 1 Facultad de Ciencias Bioquímicas y Farmacéuticas, Universidad Nacional de Rosario, Rosario, Argentina; 2 Instituto de Física Rosario (IFIR-CONICET-UNR), Rosario, Argentina; Portland State University, Portland, Oregon, USA

**Keywords:** bacteriophage, genome analysis, *Microbacterium foliorum*, DNA sequencing

## Abstract

In this work, we report the discovery and characterization of Garey24, a bacteriophage that forms medium-size plaques with halo rings isolated from a soil sample in Funes, Argentina. Its 41,522 bp circularly permuted genome contains 63 putative protein-coding genes. Based on gene content similarity, Garey24 was assigned to subcluster EA1.

## ANNOUNCEMENT

Bacteriophages are highly diverse and widely distributed viruses that infect bacteria. Studies on bacteriophages have increased due to their potential applications as therapeutic agents against antibiotic-resistant bacteria and as tools in genetic engineering (1, 2). In this work, we report the isolation and characterization of Garey24, a phage infecting *Microbacterium foliorum*.

**Fig 1:**
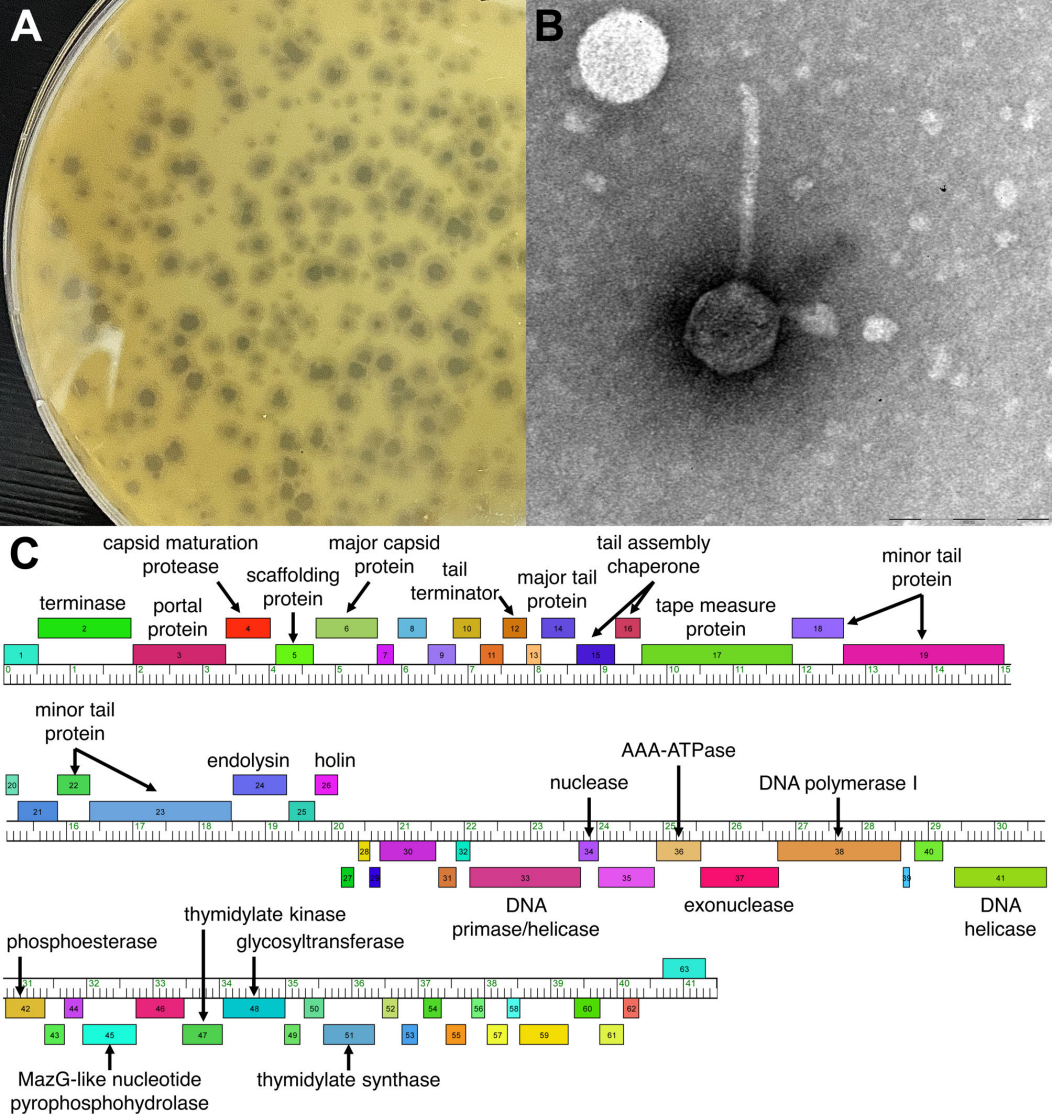
Characterization of the *Microbacterium* bacteriophage Garey24. (**A**) Garey24 forms plaques ranging from 2 to 4 mm in diameter, each surrounded by a translucent halo. (**B**) Negative-stain (1% uranyl acetate) transmission electron microscopy revealed a siphovirus morphology; an icosahedral capsid of 56 nm in diameter attached to a 127 nm-long tail (*n* = 1) Scale bar = 100 nm. Image produced by a Zeiss Libra 120 TEM with an accelerating voltage of 80 kV and processed on Olympus Soft Imaging GmbH. 5.0 (Build 1194). (**C**) Genome organization of Garey24. Genes are represented by boxes located above or below the ruler, depending on whether they are in the forward or reverse orientation, respectively. Where applicable, the putative functions of the proteins they encode are shown.

Garey24 was isolated from a soil sample collected 10–15 centimeters below the surface of the ground from a ditch in Funes, a suburban area near Rosario, Argentina (32.898028 S, 60.861667 W). The sample was suspended in a PYCa liquid medium (3) and incubated for 1 hour at 28°C. An aliquot was filtered through a 0.22-µm filter and plated in PYCa soft agar containing *Microbacterium foliorum* NRRL B-24224 as host, and further incubated at 28°C for 48 hours. A plaque with a surrounding halo (Fig. 1A) was selected and purified through three rounds of plating before being prepared as a high-titer lysate (3). Negative-staining electron microscopy showed that Garey24 has a siphovirus morphology (Fig. 1B).

Genomic DNA extraction was performed from the phage lysate using Monarch PCR and DNA cleanup kit. A sequencing library for the Garey24 genome was prepared using the NEB Ultra II library kit and sequenced on the Illumina MiSeq platform (v3 reagents), which resulted in 402,479 single-end 150 bp reads with 1,381× coverage. The genome was assembled using Newbler v2.9 (4), and accuracy and completeness were assessed with Consed v29 (5, 6). Garey24 genome is 41,522 bp long, circularly permuted, and presents a G + C content of 63.5%. Based on gene content similarity >35% to phages within the Actinobacteriophage database (https://phagesdb.org/), Garey24 was assigned to cluster EA, subcluster EA1 (7, 8)

Garey24 genome was auto-annotated using DNAmaster v5.23.6 (http://cobamide2.bio.pitt.edu/), Glimmer v3.02b (9), GeneMark v2.5p (10), and start sites manually refined using Starterator v508 (http://phages.wustl.edu/starterator/) and Phamerator (11). These analyses revealed 63 protein-coding regions, 27 of which could be functionally assigned using BLASTP v2.14.0 (against PhagesDB and NCBI nonredundant databases) (12) and HHPRed (PDB mmCIF70, Pfam-A, and NCBI Conserved Domain databases) (13) (Fig. 1C). Furthermore, one membrane protein was identified using TMHMM v2.0 (14) and SOSUI v1.11 (15), and no tRNA genes were predicted using Aragorn v1.2.41 (16) and tRNAscan-SE v2.0 (17). Default settings were used for all programs.

Consistent with the genomic organization of the EA cluster (18), the Garey24 genome is divided into two large sets of genes. The left arm contains genes predicted to be involved in virion particle formation, assembly, and release from the host (i.e., major capsid protein, major and minor tail protein, tape measure protein, portal protein, endolysin, and holin), while the right arm contains genes related to DNA metabolism such as DNA polymerase I, DNA primase/helicase, and nucleases. No immunity repressor or integrase functions were identified, suggesting that Garey24 is an obligate lytic bacteriophage.

Although the presence of halos surrounding plaques suggests depolymerase activity (19), genome analysis failed to identify genes coding for this function. Further research is required to establish the origin of halos in Garey24.

## Data Availability

Garey24 is available at GenBank with Accession No. OR521084 and Sequence Read Archive (SRA) No. SRR26666496.
